# Evolutionary rates at codon sites may be used to align sequences and infer protein domain function

**DOI:** 10.1186/1471-2105-11-151

**Published:** 2010-03-24

**Authors:** Pierre M Durand, Scott Hazelhurst, Theresa L Coetzer

**Affiliations:** 1 Evolutionary Medicine Unit, University of the Witwatersrand and National Health Laboratory Service, Johannesburg, South Africa; 2 Plasmodium Molecular Research Unit, Department of Molecular Medicine and Haematology, University of the Witwatersrand and National Health Laboratory Service, Johannesburg, South Africa; 3 Department of Ecology and Evolutionary Biology, University of Arizona, Tucson, USA; 4 School of Electrical and Information Engineering, University of the Witwatersrand, Johannesburg, South Africa

## Abstract

**Background:**

Sequence alignments form part of many investigations in molecular biology, including the determination of phylogenetic relationships, the prediction of protein structure and function, and the measurement of evolutionary rates. However, to obtain meaningful results, a significant degree of sequence similarity is required to ensure that the alignments are accurate and the inferences correct. Limitations arise when sequence similarity is low, which is particularly problematic when working with fast-evolving genes, evolutionary distant taxa, genomes with nucleotide biases, and cases of convergent evolution.

**Results:**

A novel approach was conceptualized to address the "low sequence similarity" alignment problem. We developed an alignment algorithm termed FIRE (***F***unctional ***I***nference using the ***R***ates of ***E***volution), which aligns sequences using the evolutionary rate at codon sites, as measured by the *dN*/*dS *ratio, rather than nucleotide or amino acid residues. FIRE was used to test the hypotheses that evolutionary rates can be used to align sequences and that the alignments may be used to infer protein domain function. Using a range of test data, we found that aligning domains based on evolutionary rates was possible even when sequence similarity was very low (for example, antibody variable regions). Furthermore, the alignment has the potential to infer protein domain function, indicating that domains with similar functions are subject to similar evolutionary constraints. These data suggest that an evolutionary rate-based approach to sequence analysis (particularly when combined with structural data) may be used to study cases of convergent evolution or when sequences have very low similarity. However, when aligning homologous gene sets with sequence similarity, FIRE did not perform as well as the best traditional alignment algorithms indicating that the conventional approach of aligning residues as opposed to evolutionary rates remains the method of choice in these cases.

**Conclusions:**

FIRE provides proof of concept that it is possible to align sequences and infer domain function by using evolutionary rates rather than residue similarity. This represents a new approach to sequence analysis with a wide range of potential applications in molecular biology.

## Background

Investigations in molecular biology frequently require the analysis of sequence alignments and several methods are available for this purpose. Once a correct alignment is obtained, inferences may be made concerning phylogenetic relationships and putative functions [1]. A fundamental problem arises when accurate sequence alignments cannot be obtained due to poor similarity, which may occur with homologous or analogous genes [2]. Homologous genes, comprising orthologs (arising from speciation events) and paralogs (arising from gene duplication events) share common ancestry; however, sequence similarity may be low when they are rapidly evolving, evolutionary distant, or the sequences have significant nucleotide biases. Analogous genes have similar functions, but arise from convergent evolution and the absence of shared ancestry means there is little or no sequence similarity [3].

To address the limitation of poor sequence similarity in homologous or analogous sequences, a novel alignment strategy was conceptualized and the FIRE (***F***unctional ***I***nference using ***R***ates of ***E***volution) algorithm developed. This method uses the evolutionary rate at codon sites, rather than individual residues, to align sequences. Evolutionary pressures are inferred from the parameter *ω *(ratio of non-synonymous (*dN*) to synonymous (*dS*) substitutions, corrected for opportunity) [4], which is typically used to investigate Darwinian selection at the molecular level. A non-synonymous rate significantly greater that the synonymous rate, *ω *(*dN/dS*) > 1, reflects positive selection, while neutral and purifying selection are inferred when *ω *= 1, and *ω <*1, respectively. The evolutionary rate may vary across whole coding sequences, at individual codons within a sequence or along branches within a phylogenetic tree and numerous evolutionary models and software statistical packages for performing the analyses are available. For a recent overview of the subject see [5]. The method reported here makes use of the evolutionary rate at codon sites to align sequences and demonstrates the potential to infer protein domain function in sequences that are subject to similar evolutionary constraints.

## Results and Discussion

### Conceptualization

The aim of this study was to address the limitation of poor similarity when performing sequence alignments. The traditional approach of using the positional homology of residues to align sequences was therefore abandoned and the parameter *ω *employed instead. The question we asked is: can the selective pressures acting at codon sites across coding sequences, and not residue positional homology, be used to perform alignments? To investigate this question, we aligned homologous domains (orthologous and paralogous data sets), which typically have similar functions, using *ω *values at codon sites across the sequences. Next, if sequences with similar functions can be aligned using evolutionary rates, we tested the hypothesis that this approach may be used to infer protein domain function in the absence of significant sequence similarity. Domains with poor sequence similarity but similar function (such as the antibody data sets) were employed for this purpose.

### Algorithm

The FIRE algorithm was developed in order to perform a pairwise alignment using *ω *MLEs (maximum likelihood estimates). The *ω *MLEs at codon sites were obtained from multiple sequence alignments (MSAs) of closely related orthologous sequences (see Methods below for details) and FIRE is therefore, in essence, aligning two MSAs or clades. FIRE was modified from the Needleman-Wunsch algorithm [6] and finds the pairwise alignment using the codon alignment (based on *ω *MLEs) to maximize the similarity metric. A codon score, *cs*, measures the similarity between two aligned *ω *values in the range [0,1]. The maximum difference between two *ω *values is capped to *ω*_*max *_and is parameterized - we chose 1.5 as a default, since it is biologically more meaningful to identify sites under positive selection than to emphasize the absolute values of sites with *ω*>1. Thus, *cs*(*ω1*, *ω2*) = 0, if |*ω1-ω2|*>*ω*_*max *_and *cs*(*ω1*, *ω2*) = 1-(|*ω1-ω2|*)/*ω*_*max *_otherwise. The FIRE score is the sum of the *cs *scores over all aligned codon pairs, normalized for sequence length by dividing the FIRE score by the number of codons in the longer sequence. The opening and extension gap penalties are parameterized and the defaults of 0.5 and 0.05, respectively, were used for the analyses in this study. The FIRE algorithm produces a normalized score, percentage similarity plot, histogram listing the number of codons in the alignment with similar scores per decile, and an alignment of the amino acid sequences. The FIRE software and a User Information file providing further details are freely available at http://dept.ee.wits.ac.za/~scott/fire and are attached as additional files 1 and 2.

### Testing

#### Data sets

The Bayes Empirical Bayes (model M2) or Naïve Empirical Bayes (model M3) posterior mean *ω *MLEs at codon sites were obtained for 15 data sets using the PAML (Phylogenetic Analysis using Maximum Likelihood) v4.0 software [7] and a FIRE alignment of each data set with every other set was performed (225 alignments for each model). Data sets included the following domains: (i) a highly conserved transcription factor MYB1 DNA-binding domain (DBD) [8]; (ii) MYB2, a paralog of MYB1; (iii) a conserved tumor suppressor p53 DBD [9]; (iv) a metabolic enzyme glycerol kinase (GK) [10]; and (v) light chain antibody variable regions [11]. Variations in the following parameters were present across data sets and did not adversely affect PAML or FIRE analyses: domain length (90-504 codons), *ω *MLE range (0-9), *dN *range (0.2-55.0), and paralogous sequences (metazoan MYB1 and MYB2). The number of sequences per data set ranged from 4-12. The data sets with low sequence number were used to examine the effect of this on FIRE outputs, and as expected, sets with fewer sequences produced less accurate *ω *MLEs and decreased FIRE reliability. Sequence divergence (*dS *value across the tree) varied from 2.1 to 48.1, which is within PAML suggested limits of <50 [7]. An exception was the protozoan MYB1 set (*dS *= 216.1); however, it is unlikely this led to erroneous results since high *dS *values falsely elevate *ω *values and in this set all the *ω *MLEs were <0.3.

#### Data analysis

**Figure 1:**
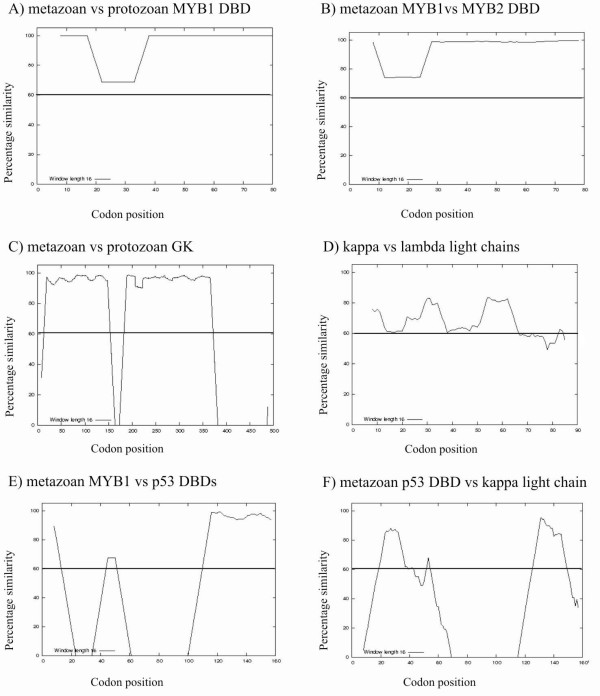
**FIRE plots**. Plots represent the pairwise alignment of *ω *MLEs at codon sites with FIRE, recorded as a percent similarity between the two values. Corresponding FIRE scores and alignments are in TABLE 1 and additional file 3, respectively. A sliding window of 16 codons was used and the percent similarity is the average over the window. (A) conserved orthologous metazoan and protozoan MYB1 DBDs; (B) conserved paralogous metazoan MYB1 and MYB2 DBDs; (C) conserved metazoan and protozoan GK; (D) κ and λ light chain antibodies; (E) metazoan MYB1 and p53 DBDs; and (F) p53 DBD and κ light chain antibody. The sequence sets used in plots E and F have no functional similarity and represent negative controls. The 60% similarity cut-off value is indicated by a solid line. DBD = DNA-binding domain; GK = glycerol kinase.

The results of salient examples are discussed. FIRE scores and similarity plots are provided in Table 1 and Figure 1, respectively, and the corresponding FIRE alignments are documented in additional file 3. The results presented were obtained for *ω *MLEs obtained from PAML analyses under model M3 (NSsites = 3). In general, FIRE results for *ω *MLEs with model M3 were the same or better than M2, the likely reason being that *ω *MLEs under M3 are less constrained.

**Table 1:** FIRE scores

Set	Data sets aligned	^#^**ω**	FIRE score
**1**	*metazoan MYB1 and protozoan MYB1	ω ≤ 0.2	0.93

**2**	*metazoan MYB1 and metazoan MYB2	ω ≤ 0.3	0.94

**3**	protozoan MYB1 and metazoan MYB2	ω ≤ 0.3	0.99

**4**	*metazoan GK and protozoan GK	ω ≤ 1.3	^+^0.62

**5**	^€^κ light chain VR and κ light chain VR	ω ≤ 7.0	0.66

**6**	*κ light chain VR and λ light chain VR	ω ≤ 8.2	0.65

**7**	*metazoan MYB1 and metazoan p53	ω ≤ 1.3	0.45

**8**	metazoan MYB1 and metazoan GK	ω ≤ 1.3	0.09

**9**	κ light chain VR and metazoan p53	ω ≤ 7.0	0.29

**10**	*metazoan p53 and λ light chain VR	ω ≤ 8.2	0.32

The results of 10 FIRE alignments of the *ω *MLEs derived from two sequence sets are shown. The range of *ω *MLEs at codon sites (^#^) includes values for both data sets, and was taken from model M3 results. FIRE plots and alignments for sets marked with asterisk (*) are provided in Figure 1 and additional file 3, respectively. The two κ data sets (labeled ^€^) represent different κ sequences. Metazoan and protozoan GK data sets differed by >100 codons and therefore produced a relatively low FIRE alignment score (^+^). DNA-binding domains were used for MYB and p53 alignments. Sets 7-10 are negative controls. GK = glycerol kinase; VR = variable region.

**Figure 2:**
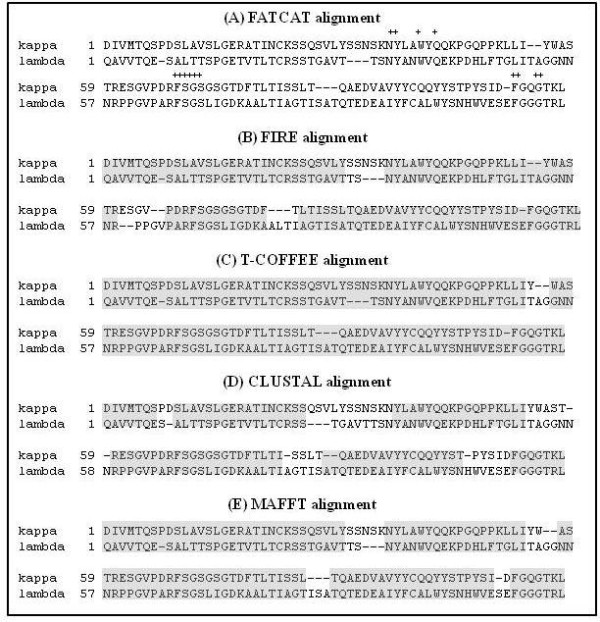
**FIRE, T-Coffee, ClustalW and MAFFT MSAs**. The alignments generated by (A) FATCAT, (B) FIRE, (C) T-Coffee, (D) ClustalW and (E) MAFFT algorithms for kappa and lambda antibody variable regions (data set 6 in Table 1) are displayed. Only sequences corresponding to the two structure files in the FATCAT alignment and the representative sequences from the two clades aligned by FIRE are shown for each of the other three MSAs. Using the FATCAT alignment as an independent standard-of-truth reference, correctly aligned residue pairs in the other four MSAs were identified (shaded regions). Overall, T-Coffee and MAFFT produced the most accurate alignments, however, FIRE performed better than ClustalW demonstrating the viability of using an evolutionary rates-based approach to sequence analysis when sequence similarity is low. In addition, the short stretches of conserved amino acids (indicated by +) inflate the performances of the three homology-based methods relative to FIRE (see text for discussion).

Following normalization for sequence length, homologous domains with similar functions produced FIRE scores >0.60 and FIRE plots with the majority of codon similarities >60%. For example, alignments for two orthologous sets (metazoan MYB1/protozoan MYB1) and two paralogous sets (metazoan MYB1/MYB2) produced scores of 0.93 and 0.94, respectively, and similarity plots nearing 100% over most of the sequence. The metazoan GK/protozoan GK alignment provided a comparison of two orthologous sets with a greater range in *ω *MLEs, significantly different sequence lengths (>100 codons) and non-contiguous evolutionary conserved codons [12]. The difference in sequence length is responsible for the relatively low score (0.62), which is reflected by the gaps in the plot (Figure 2C). Removing the gaps prior to performing a FIRE alignment increased the score to 0.89.

Conserved domains with dissimilar functions produced poor alignments, for example the metazoan MYB1/GK alignment (FIRE score = 0.09). The MYB1/p53 DBD alignment provided an interesting test case. Both are transcription factors, however, the domains are implicated in very different biological functions and, according to our hypothesis, this difference should result in a poor FIRE alignment. This was indeed the case (FIRE score = 0.45). We did, however, note that FIRE produced false positives when two unrelated highly conserved domains (*ω *MLEs <0.3 across the entire sequence) of similar lengths were aligned (data not shown). Including other computational methods such as structure determination would be valuable to identify these cases.

The effect of sites under strong positive selection on this approach was observed from alignments that included antibody sets. As a result of positive selection, antibody sequences of the variable region demonstrate poor sequence similarity. Despite this, the FIRE scores (>0.60), plots and alignments between the two κ sets, and the κ and λ sets suggested that these domains are under similar evolutionary pressures, which correlates with their similar functions. Alignments of the κ and λ antibody sets with any of the domains that are functionally unrelated, for example MYB1, GK or p53, produced poor FIRE results.

#### FIRE performance

To compare the performance of FIRE against the more conventional ClustalW [13], MAFFT [14] and structure-based T-Coffee [15] algorithms for the data sets in Table 1, we employed sequence-structure alignments (based on known 3D molecular structures) as an independent standard of truth. FATCAT (***F***lexible structure ***A***lignmen ***T ***by ***C***haining ***A***ligned fragment pairs allowing ***T***wists) [16] and DALI (***D***istance matrix ***Ali***gnment program) [17] algorithms, which differ in their treatments of flexible structures, were used to align data sets 1-6 (negative control data sets cannot be aligned due to different 3D structures) based on structures extracted from the worldwide Protein Data Bank [18]. As a measure of performance, the proportions of correctly aligned residue pairs (including gaps) obtained by FIRE, T-Coffee, ClustalW and MAFFT alignments were determined using FATCAT or DALI as the reference alignment. For example, in data set 1 FATCAT aligned 54 pairs of residues, and using this as the reference alignment, the proportions correctly aligned by FIRE, T-Coffee, ClustalW and MAFFT were 0.87, 1.00, 0.99 and 1.00, respectively. Irrespective of whether FATCAT or DALI was used as the reference alignment, FIRE, T-Coffee, ClustalW and MAFFT produced similar performances. Results with FATCAT as the reference alignment are summarized in Table 2 and data set 6 is provided as an alignment example in Figure 2.

**Table 2:** FIRE, T-Coffee, ClustalW and MAFFT performances

Set	FATCAT residue pairs	FIRE performance	T-Coffee performance	ClustalW performance	MAFFT performance
**1**	54	0.87	1.00	0.99	1.00

**2**	94	0.57	0.96	0.84	0.97

**3**	137	0.83	0.97	0.83	0.96

*4	-	0.69	1.00	0.68	0.94

**5**	103	0.83	0.98	0.71	0.88

**6**	108	0.87	0.97	0.73	0.87

Performances of the FIRE, T-Coffee, ClustalW and MAFFT algorithms were measured by determining the proportion of correctly aligned residue pairs using FATCAT and DALI structure-based alignments as a reference. FIRE is independent of homology and performed better than ClustalW for data sets 5 and 6 (antibody variable regions), illustrating the value of using this approach when sequence similarities are low. This independence from residues in the sequence may also lead to relatively poor FIRE performance when sequence similarity is high, for example set 2. T-Coffee and MAFFT performed best overall (although see text for further discussion). The PDB structure files included in FATCAT, DALI and T-Coffee algorithms are set 1: 2DIM, 2K9N; set 2: 2DIM, 2DIN; set 3: 2YUM, 2K9N; set 4: 1BO5; set 5: 5LVE, 1QP1; set 6: 1LVE, 1NC4. *Due to a lack of structural data for set 4, the FUGUE threading algorithm [23] was used to generate a reference structure alignment from the *E. histolytica *sequence (XM_650121.1) using *E. coli *glycerol kinase (PDB IB: 1BO5) as a template. For all alignments, FATCAT and DALI produced similar results and only the FATCAT data are shown.

The T-Coffee and MAFFT algorithms performed the same or better than FIRE or ClustalW for all data sets. The T-Coffee performance is unsurprising since (i) the same structure files used by FATCAT and DALI were included in the T-Coffee algorithm, and (ii) it is well known that structure-based alignments or a combination of structural information with other approaches (such as T-Coffee which combines structural data with homology-based methods) produce the most accurate alignments when sequence similarity is low [19]. Although T-Coffee and MAFFT are state-of-the art methods and known to perform well, it is worth noting that in data sets 5 and 6, the performances of the three homology-based algorithms (T-Coffee, MAFFT and ClustalW) may be inflated relative to FIRE due to the presence of short stretches of conserved residues involved in stabilizing the tertiary structures of the antibody light chains. It was also observed that FIRE performed better than ClustalW in these same data sets, demonstrating the value of aligning sequences based on evolutionary rates when sequence similarity is low. One shortcoming of the FIRE algorithm is that the performance may actually decrease when sequence similarity is high. The likely reason is that if the two sequences being aligned share a long stretch of very low *ω *MLEs, such as occurs in highly conserved domains like the MYB transcription factor in data set 2, there is a risk that a gap (possibly due to an insertion/deletion elsewhere in one of the sequences) is introduced causing a misalignment over the conserved region.

The main findings from these results indicate that it is conceptually and methodologically possible to align functionally similar domains accurately using the evolutionary rate at codon sites. In addition, good alignment scores were only obtained for sequences coding for similar functions, indicating that domains with similar functions are subject to similar evolutionary constraints. This suggests that the FIRE approach may be valuable for inferring domain function in situations such as convergent evolution or when sequences are highly divergent. However, it was noted that for homologous genes where there is some sequence similarity, FIRE was not as accurate as MAFFT or T-Coffee. In these cases, the conventional algorithms remain the method of choice.

### Implementation

The value of the FIRE approach currently lies in its ability to align sequences independent of residue similarity. This is helpful for analyzing sequences with poor similarity, which typically occurs with evolutionary distant genes, convergent evolution and sequences with extreme nucleotide biases [20]. It is known that structure-based methods are also valuable in these circumstances and it is likely, therefore, that a combination of the two approaches will offer the best strategy. Structural information may also be valuable for eliminating false positives and negatives produced by FIRE. Furthermore, all components of the FIRE output: normalized scores, plots, alignments and histograms should be evaluated in their biological context. Further experimentation and subsequent refinements to the FIRE algorithm will lead to improvements in method sensitivity and specificity.

## Conclusions

FIRE provides proof of concept that it is possible to align sequences and infer domain function by using evolutionary rates. It complements the arsenal of available computational methods and represents a new approach to sequence analyses with a wide range of potential applications in molecular biology.

## Methods

### MLEs of the ω parameter

Coding sequences were extracted from NCBI http://www.ncbi.nlm.nih.gov and PlasmoDB v5.5 http://www.plasmodb.org databases. MSAs were performed with MAFFT [14] and phylogenetic trees were constructed with ClustalW2 [21] and PAUP* [22]. MSAs and phylogenetic guide trees were processed with PAML 4 (codeml algorithm, F3 × 4 codon model, Model = 0, NSsites = 2 and 3) [7] to obtain *ω *MLEs at codon sites. Each data set (comprising a list of *ω *MLEs) was aligned with every other data set with the program FIRE, which was specifically developed for this purpose (see "*algorithm*" section above). A list of accession numbers, sequence details, and PAML sequence and tree files are available from the corresponding author.

### FIRE analysis

FIRE uses the rst output files from a PAML analysis (using either NSsites 2 or 3) to extract the *ω *MLEs and perform an alignment. Two examples of the PAML rst and mlc raw output files of the protozoan GK and λ light chain variable regions data set are provided in additional file 4. The rst files provide *ω *MLEs and mlc files provide statistical details regarding the analysis, for example *dN*, *dS *and kappa (transition/transversion ratio) values. The FIRE results for all data sets aligned with each other are available from the corresponding author.

### T-Coffee, ClustalW and MAFFT MSAs

T-Coffee, ClustalW and MAFFT analyses were performed using online servers at the Swiss Institute of Bioinformatics http://tcoffee.vital-it.ch/cgi-bin/Tcoffee/tcoffee_cgi/index.cgi, the European Bioinformatics Institute http://www.ebi.ac.uk/Tools/ and the MAFFT homepage http://align.bmr.kyushu-u.ac.jp/mafft/software, respectively. The T-Coffee advanced algorithm combined a ClustalW alignment with the available PDB structure files, which were selected based on the corresponding data set sequence files and downloaded from the World Wide Protein Databank http://www.wwpdb.org. The *E. coli *crystal structure PDB file (ID: 1BO5) was used for a T-Coffee analysis of the glycerol kinase set due to a lack of metazoan and protozoan structural data. Default settings were used for ClustalW2 alignments. For the MAFFT analysis, the E-INS-i algorithm was used.

### FATCAT and DALI alignments

Sequence-structure alignments were performed with FATCAT http://fatcat.burnham.org and DALI http://ekhidna.biocenter.helsinki.fi/dali_server online servers. The two PDB structures used for each alignment are representative sequences taken from the two clades being aligned with FIRE. The same structures were included in T-Coffee alignments. Due to a lack of structural data for the GK sequence set (data set 4), the FUGUE threading algorithm [23] was used to generate a second structure for *E. histolytica *GK (XM_650121.1) with the *E. coli *crystal structure (PBD ID: 1BO5) as a template. FATCAT, DALI and FUGUE structure alignments were used as reference alignments to which FIRE, T-Coffee, ClustalW and MAFFT alignments were compared.

## Note added in proof

Our results complement a recent publication by S.L. Kosakovsky Pond *et al. *("Evolutionary Fingerprinting of Genes", Mol Biol Evol 2010, 27:520-536), which demonstrated that probability distributions of evolutionary rates in coding sequences may be used as identifiers of genes. Furthermore, using rapidly evolving RNA viruses as test data, they found that genes within the same functional group have similar evolutionary fingerprints. The findings presented by Kosakovsky Pond *et al. *and the data in this manuscript suggest that the molecular signatures left behind by evolution represent a tier of information that is untapped by current sequence analysis methods.

## Authors' contributions

PMD conceptualized the study. PMD and SH performed the experiments. SH scripted the FIRE algorithm. PMD, SH and TLC analyzed the results and wrote the manuscript. All authors read and approved the final manuscript.

## Supplementary Material

Additional file 1 **FIRE script**. Python code for the FIRE algorithm. Click here for file

Additional file 2 **FIRE User Information File**. Information for users of FIRE algorithm. Click here for file

Additional file 3 **FIRE alignments**. Alignments correspond to the plots in Figure 1. Each alignment is presented in fasta and interleaved formats. For interleaved format: residues are shaded as identical (black) or similar (gray), except for (D), which is shaded as in Figure 2. (A) highly conserved metazoan and protozoan MYB1 DBDs; (B) conserved paralogous metazoan MYB1 and MYB2 DBDs; (C) conserved metazoan and protozoan GK; (D) κ and λ light chain antibodies; (E) metazoan MYB1 and p53 DBDs; and (F) p53 DBD and κ light chain antibody. The sequence sets used in alignments E and F have no functional similarity and represent negative controls. Click here for file

Additional file 4 **PAML rst and mlc output examples**. Two examples of the raw data PAML 4.0 mlc and rst output files for the protozoan GK and lambda light chain antibody data sets. Click here for file
