# Supplementary material for: Evolutionary rates at codon sites may be used to align sequences and infer protein domain function
Source: BMC Bioinformatics. 2010 Mar 24;11:151. doi: 10.1186/1471-2105-11-151 (PMC2851608; doi:10.1186/1471-2105-11-151)
Supplement: Additional file 3 — FIRE alignments. Alignments correspond to the plots in Figure 1. Each alignment is presented in fasta and interleaved formats. For interleaved format: residues are shaded as identical (black) or similar (gray), except for (D), which is shaded as in Figure 2. (A) highly conserved metazoan and protozoan MYB1 DBDs; (B) conserved paralogous metazoan MYB1 and MYB2 DBDs; (C) conserved metazoan and protozoan GK; (D) κ and λ light chain antibodies; (E) metazoan MYB1 and p53 DBDs; and (F) p53 DBD and κ light chain antibody. The sequence sets used in alignments E and F have no functional similarity and represent negative controls. [file 1471-2105-11-151-S3.DOC]

**FIRE alignments.**

Alignments correspond to the plots in Figure 1. Each alignment is presented in fasta and interleaved formats. For interleaved format: residues are shaded as identical (black) or similar (gray). (A) highly conserved metazoan and protozoan MYB1 DBDs; (B) conserved paralogous metazoan MYB1 and MYB2 DBDs; (C) conserved metazoan and protozoan GK; (D) κ and λ light chain antibodies; (E) metazoan MYB1 and p53 DBDs; and (F) p53 DBD and κ light chain antibody. The sequence sets used in alignments E and F have no functional similarity and represent negative controls.

A)

>MYB1meta

KGGAWKNTEDAVSKYGKNQWARISSLLVRKTP.....KQCKARWYEWIDPSIKKTEWSRE

EDEKLLHLAKLLPTQWRTIAPIVGRTATQCLERYQ

>MYB1proto

KGGIWKNCEDEVLKAAVMKYGLNNWSRVASLLVRKSAKQCKARWYEWLDPSVKKTEWSKE

EEEKLLHLAKLFPTQWRTIAPIVGRTAQQCLEHYE

**10 20 30 40 50 60**

**....|....| ....|....| ....|....| ....|....| ....|....| ....|....|**

**MYB1meta**  KGGAWKNTED AVSKYGKNQW ARISSLLVRK TP.....KQC KARWYEWIDP SIKKTEWSRE

**MYB1proto** KGGIWKNCED EVLKAAVMKY GLNNWSRVAS LLVRKSAKQC KARWYEWLDP SVKKTEWSKE

**70 80 90**

**....|....| ....|....| ....|....| ....|**

**MYB1meta**  EDEKLLHLAK LLPTQWRTIA PIVGRTATQC LERYQ

**MYB1proto** EEEKLLHLAK LFPTQWRTIA PIVGRTAQQC LEHYE

B)

>MYB1meta

KGGAWKNTEDAVSKYGKNQWARI....SSLLVRKTPKQCKARWYEWIDPSIKKTEWSREE

DEKLLHLAKLLPTQWRTIAPIVGRTATQCLERYQ

>MYB2meta

EELMNYGSNSDNEDTQVCDKDLDSPELKSGKTSIGAVRWSKSEDVLLKQLVETHGENWEI

IGPHFKDRLEQQVQQRWAKVLNPELIKGPWTRDE

**10 20 30 40 50 60**

**....|....| ....|....| ....|....| ....|....| ....|....| ....|....|**

**MYB1meta** KGGAWKNTED AVSKYGKNQW ARI....SSL LVRKTPKQCK ARWYEWIDPS IKKTEWSREE

**MYB2meta** EELMNYGSNS DNEDTQVCDK DLDSPELKSG KTSIGAVRWS KSEDVLLKQL VETHGENWEI

**70 80 90**

**....|....| ....|....| ....|....| ....**

**MYB1meta** DEKLLHLAKL LPTQWRTIAP IVGRTATQCL ERYQ

**MYB2meta** IGPHFKDRLE QQVQQRWAKV LNPELIKGPW TRDE

C)

>GKmeta

MAAAKKAPLVGAVDQGTSSTRFLVFNSKTAELLSHHQVEIKQEFPREGWVEQDPKEILQS

VYECIEKTCEKLGQLNIDISNIKAIGVSNQRETTVVWDKVTGEPLYNAVVWLDLRTQSTV

ENLSKRIPGNNNFVKSKTGLPLSTYFSAVKLRWLLDNVKKVQEAVEENRALFGTIDSWLI

WSLTGGIVHCTDVTNASRTMLFNIHSLEWDKELCEFFGIPMEILPNVRSSSEIYGLMKAG

ALEGVPISGCLGDQSAALVGQMCFQDGQAKNTYGTGCFLLCNTGHKCVFSEHGLLTTVAY

KLGRDKVYYALEGSVAIAGAVIRWLRDNLGIIKSSEEIEKLAKEVGTSYGCYFVPAFSGL

YAPYWEPSARGIICGLTQFTNKCHIAFAALEAVCFQTREILDAMNRDCGIPLSHLQVDGG

MTSNKILMQLQADILYIPVVKPSMPETTALGAAMAAGAGVGWSLEPEDLSAVTMFEPQIN

AEESEIRYSTWKKAVMKSIGWVTT

>GKproto

MKY...............IIDQGTTSTRVILFDEKCQSIHTEQEEFDFPHPGWVEQDPEV

IYTSVVNLMKKCLVNTGINKDIAAIGITNQRETTVMWDKRTGKPIYNAIVWQSKQSGNET

SYLMEKIFQSKTGLVLNPYFSASKIMWIFNNVEGAKALAEEGV.................

........NLTGGHTDISNAARTLLFNIYEKKWDDELLAKT.NIPKSILPIVKQSSDDFG

IVSTIQEFHITGVAGDQQASLFGHGSPIGGCKSTYGTGCFVVKNIGDTIKEIPKGLLATV

GWEINGKITYALEGTVMTAGAALKWIRDIGILKDYNEISKIVTSKNGGVYFVPAFQGLGT

PYWDDDVRGIIVGLTSGTGKG.......................................

............................................................

.....................ELV

**10 20 30 40 50 60**

**....|....| ....|....| ....|....| ....|....| ....|....| ....|....|**

**GKmeta**  MAAAKKAPLV GAVDQGTSST RFLVFNSKTA ELLSHHQVEI KQEFPREGWV EQDPKEILQS

**GKproto** MKY....... ........II DQGTTSTRVI LFDEKCQSIH TEQEEFDFPH PGWVEQDPEV

**70 80 90 100 110 120**

**....|....| ....|....| ....|....| ....|....| ....|....| ....|....|**

**GKmeta**  VYECIEKTCE KLGQLNIDIS NIKAIGVSNQ RETTVVWDKV TGEPLYNAVV WLDLRTQSTV

**GKproto** IYTSVVNLMK KCLVNTGINK DIAAIGITNQ RETTVMWDKR TGKPIYNAIV WQSKQSGNET

**130 140 150 160 170 180**

**....|....| ....|....| ....|....| ....|....| ....|....| ....|....|**

**GKmeta**  ENLSKRIPGN NNFVKSKTGL PLSTYFSAVK LRWLLDNVKK VQEAVEENRA LFGTIDSWLI

**GKproto** SYLMEKIFQS KTGLVLNPYF SASKIMWIFN NVEGAKALAE EGV....... ..........

**190 200 210 220 230 240**

**....|....| ....|....| ....|....| ....|....| ....|....| ....|....|**

**GKmeta**  WSLTGGIVHC TDVTNASRTM LFNIHSLEWD KELCEFFGIP MEILPNVRSS SEIYGLMKAG

**GKproto** ........NL TGGHTDISNA ARTLLFNIYE KKWDDELLAK T.NIPKSILP IVKQSSDDFG

**250 260 270 280 290 300**

**....|....| ....|....| ....|....| ....|....| ....|....| ....|....|**

**GKmeta**  ALEGVPISGC LGDQSAALVG QMCFQDGQAK NTYGTGCFLL CNTGHKCVFS EHGLLTTVAY

**GKproto** IVSTIQEFHI TGVAGDQQAS LFGHGSPIGG CKSTYGTGCF VVKNIGDTIK EIPKGLLATV

**310 320 330 340 350 360**

**....|....| ....|....| ....|....| ....|....| ....|....| ....|....|**

**GKmeta**  KLGRDKVYYA LEGSVAIAGA VIRWLRDNLG IIKSSEEIEK LAKEVGTSYG CYFVPAFSGL

**GKproto** GWEINGKITY ALEGTVMTAG AALKWIRDIG ILKDYNEISK IVTSKNGGVY FVPAFQGLGT

**370 380 390 400 410 420**

**....|....| ....|....| ....|....| ....|....| ....|....| ....|....|**

**GKmeta**  YAPYWEPSAR GIICGLTQFT NKCHIAFAAL EAVCFQTREI LDAMNRDCGI PLSHLQVDGG

**GKproto** PYWDDDVRGI IVGLTSGTGK G......... .......... .......... ..........

**430 440 450 460 470 480**

**....|....| ....|....| ....|....| ....|....| ....|....| ....|....|**

**GKmeta**  MTSNKILMQL QADILYIPVV KPSMPETTAL GAAMAAGAGV GWSLEPEDLS AVTMFEPQIN

**GKproto** .......... .......... .......... .......... .......... ..........

**490 500**

**....|....| ....|....| ....**

**GKmeta**  AEESEIRYST WKKAVMKSIG WVTT

**GKproto** .......... .......... .ELV

D)

This data set corresponds to the FIRE alignment in Figure 2. The shading used here as the same as in Figure 2.

>kappa

DIVMTQSPDSLAVSLGERATINCKSSQSVLYSSNSKNYLAWYQQKPGQPPKLLI--YWASTRESGV--PDRFSGSGSGTDF---TLTISSLTQAEDVAVYYCQQYYSTPYSID-FGQGTKL

>lambda

QAVVTQE-SALTTSPGETVTLTCRSSTGAVTTS---NYANWVQEKPDHLFTGLITAGGNNNR--PPGVPARFSGSLIGDKAALTIAGTISATQTEDEAIYFCALWYSNHWVESEFGGGTRL

kappa 1 DIVMTQSPDSLAVSLGERATINCKSSQSVLYSSNSKNYLAWYQQKPGQPPKLLI--YWAS

lambda 1 QAVVTQE-SALTTSPGETVTLTCRSSTGAVTTS---NYANWVQEKPDHLFTGLITAGGNN

kappa 59 TRESGV--PDRFSGSGSGTDF---TLTISSLTQAEDVAVYYCQQYYSTPYSID-FGQGTKL

lambda 57 NR--PPGVPARFSGSLIGDKAALTIAGTISATQTEDEAIYFCALWYSNHWVESEFGGGTRL

E)

>MYB1meta

KGGAWKNTEDAVSKYGKNQWARIS........................SLLVRKTPKQCK

A...............................................RWYEWIDPSIKK

TEWSREEDEKLLHLAKLLPTQWRTIAPIVGRTATQCLERYQ............

>p53meta

SVTCTYSPDLNKMFCQLAKTCPVQLWVDSTPPPGSRVRAMAIYKQSQHMTEVVRRCPHHE

RCSSDGLAPPQHLIRVEGNLRVEYSDDRNTFRHSVVVPYEPPEVGSDCTTIHYNYMCNSS

CMGGMNRRPILTIITLEDSSGNLLGRNSFEVRVCACPGRDRRTEEENFRKKGE

**10 20 30 40 50 60**

**....|....| ....|....| ....|....| ....|....| ....|....| ....|....|**

**MYB1meta** KGGAWKNTED AVSKYGKNQW ARIS...... .......... ........SL LVRKTPKQCK

**p53meta**  SVTCTYSPDL NKMFCQLAKT CPVQLWVDST PPPGSRVRAM AIYKQSQHMT EVVRRCPHHE

**70 80 90 100 110 120**

**....|....| ....|....| ....|....| ....|....| ....|....| ....|....|**

**MYB1meta** A......... .......... .......... .......... ........RW YEWIDPSIKK

**p53meta**  RCSSDGLAPP QHLIRVEGNL RVEYSDDRNT FRHSVVVPYE PPEVGSDCTT IHYNYMCNSS

**130 140 150 160 170**

**....|....| ....|....| ....|....| ....|....| ....|....| ...**

**MYB1meta** TEWSREEDEK LLHLAKLLPT QWRTIAPIVG RTATQCLERY Q......... ...

**p53meta**  CMGGMNRRPI LTIITLEDSS GNLLGRNSFE VRVCACPGRD RRTEEENFRK KGE

F)

>p53

SVTCTYSPDLNKMFCQLAKTCPVQLWVDSTPPPGSRVRAMAIYKQSQHMTEVVRRCPHHE

RCSSDGLAPPQHLIRVEGNLRVEYSDDRNTFRHSVVVPYEPPEVGSDCTTIHYNYMCNSS

CMGGMNRRPILTIITLEDSSGNLLGRNSFEVRVCACPGRDRRTEEENFRKKGE

>kappa

........................SFLSASVGDRVTITCRASQGISYLAWY........Q

QKPGKAPKLLIYAASTLQSGVPSRFSGSGSGTEFTLTISSLQPEDFATYYCQQN......

.............................................SYPTVLQT

**10 20 30 40 50 60**

**....|....| ....|....| ....|....| ....|....| ....|....| ....|....|**

**p53**  SVTCTYSPDL NKMFCQLAKT CPVQLWVDST PPPGSRVRAM AIYKQSQHMT EVVRRCPHHE

**kappa** .......... .......... ....SFLSAS VGDRVTITCR ASQGISYLAW Y........Q

**70 80 90 100 110 120**

**....|....| ....|....| ....|....| ....|....| ....|....| ....|....|**

**p53**  RCSSDGLAPP QHLIRVEGNL RVEYSDDRNT FRHSVVVPYE PPEVGSDCTT IHYNYMCNSS

**kappa** QKPGKAPKLL IYAASTLQSG VPSRFSGSGS GTEFTLTISS LQPEDFATYY CQQN......

**130 140 150 160 170**

**....|....| ....|....| ....|....| ....|....| ....|....| ...**

**p53**  CMGGMNRRPI LTIITLEDSS GNLLGRNSFE VRVCACPGRD RRTEEENFRK KGE

**kappa** .......... .......... .......... .......... .....SYPTV LQT
