# Supplementary material for: Evolutionary rates at codon sites may be used to align sequences and infer protein domain function
Source: BMC Bioinformatics. 2010 Mar 24;11:151. doi: 10.1186/1471-2105-11-151 (PMC2851608; doi:10.1186/1471-2105-11-151)
Supplement: Additional file 4 — PAML rst and mlc output examples. Two examples of the raw data PAML 4.0 mlc and rst output files for the protozoan GK and lambda light chain antibody data sets. [file 1471-2105-11-151-S4.DOC]

**PAML rst and mlc output examples**

Below are 2 examples of the raw data PAML 4.0 mlc and rst output files. The mlc files provide details for the PAML analysis, for example, N, S, dN, dS, t values. The rst file contains the posterior mean Bayes Empirical Bayes omega estimate for each site.

The examples are the protozoan GK data (set 6), and the lambda light chain antibody variable region data (set 15).

1. **Protozoa GK: raw mlc file**

seed used = 650702217

Before deleting alignment gaps

12 1506

CODONML (in paml version 4, June 2007) seq.nuc Model: One dN/dS ratio

Codon frequencies: F3x4

Site-class models: PositiveSelection

ns = 12 ls = 343

TREE # 1: (1, (((2, 3), 4), (5, 6)), ((7, ((8, 9), 10)), (11, 12))); MP score: 1744

lnL(ntime: 21 np: 26): -9369.944757 +0.000000

13..1 13..14 14..15 15..16 16..2 16..3 15..4 14..17 17..5 17..6 13..18 18..19 19..7 19..20 20..21 21..8 21..9 20..10 18..22 22..11 22..12

6.701928 0.136803 2.123076 2.950274 2.179919 2.165608 6.961897 4.396248 0.189529 0.685206 3.319574 0.326382 0.805860 0.437432 0.077124 0.071235 0.062388 0.081641 0.474712 0.164732 0.284308 1.457738 0.916315 0.000000 0.051471 1.000000

Note: Branch length is defined as number of nucleotide substitutions per codon (not per neucleotide site).

tree length = 34.59588

(1: 6.701928, (((2: 2.179919, 3: 2.165608): 2.950274, 4: 6.961897): 2.123076, (5: 0.189529, 6: 0.685206): 4.396248): 0.136803, ((7: 0.805860, ((8: 0.071235, 9: 0.062388): 0.077124, 10: 0.081641): 0.437432): 0.326382, (11: 0.164732, 12: 0.284308): 0.474712): 3.319574);

(E_histolytica: 6.701928, (((T_brucei: 2.179919, L_major: 2.165608): 2.950274, G_lamblia: 6.961897): 2.123076, (T_annulata: 0.189529, T_parva: 0.685206): 4.396248): 0.136803, ((P_falciparum: 0.805860, ((P_berghei: 0.071235, P_yoelii: 0.062388): 0.077124, P_chabaudi: 0.081641): 0.437432): 0.326382, (P_knowlesi: 0.164732, P_vivax: 0.284308): 0.474712): 3.319574);

Detailed output identifying parameters

kappa (ts/tv) = 1.45774

dN/dS for site classes (K=3)

p: 0.91632 0.00000 0.08368

w: 0.05147 1.00000 1.00000

dN & dS for each branch

branch t N S dN/dS dN dS N*dN S*dS

13..1 6.702 791.0 238.0 0.1308 0.8808 6.7315 696.7 1602.0

13..14 0.137 791.0 238.0 0.1308 0.0180 0.1374 14.2 32.7

14..15 2.123 791.0 238.0 0.1308 0.2790 2.1324 220.7 507.5

15..16 2.950 791.0 238.0 0.1308 0.3877 2.9633 306.7 705.2

16..2 2.180 791.0 238.0 0.1308 0.2865 2.1895 226.6 521.1

16..3 2.166 791.0 238.0 0.1308 0.2846 2.1752 225.1 517.7

15..4 6.962 791.0 238.0 0.1308 0.9150 6.9926 723.7 1664.2

14..17 4.396 791.0 238.0 0.1308 0.5778 4.4156 457.0 1050.9

17..5 0.190 791.0 238.0 0.1308 0.0249 0.1904 19.7 45.3

17..6 0.685 791.0 238.0 0.1308 0.0901 0.6882 71.2 163.8

13..18 3.320 791.0 238.0 0.1308 0.4363 3.3342 345.1 793.5

18..19 0.326 791.0 238.0 0.1308 0.0429 0.3278 33.9 78.0

19..7 0.806 791.0 238.0 0.1308 0.1059 0.8094 83.8 192.6

19..20 0.437 791.0 238.0 0.1308 0.0575 0.4394 45.5 104.6

20..21 0.077 791.0 238.0 0.1308 0.0101 0.0775 8.0 18.4

21..8 0.071 791.0 238.0 0.1308 0.0094 0.0715 7.4 17.0

21..9 0.062 791.0 238.0 0.1308 0.0082 0.0627 6.5 14.9

20..10 0.082 791.0 238.0 0.1308 0.0107 0.0820 8.5 19.5

18..22 0.475 791.0 238.0 0.1308 0.0624 0.4768 49.4 113.5

22..11 0.165 791.0 238.0 0.1308 0.0216 0.1655 17.1 39.4

22..12 0.284 791.0 238.0 0.1308 0.0374 0.2856 29.6 68.0

1. **Protozoa GK: raw rst file**

lnL = -9369.944757

Bayes Empirical Bayes (BEB) probabilities for 3 classes (class)& postmean_w

E_histolytica used as reference

1 M 1.00000 0.00000 0.00000 ( 1) 0.050 +- 0.000

2 K 0.03551 0.69145 0.27304 ( 2) 1.105 +- 0.310

3 Y 0.63800 0.29039 0.07161 ( 1) 0.432 +- 0.530

4 I 0.99968 0.00028 0.00005 ( 1) 0.050 +- 0.019

5 I 1.00000 0.00000 0.00000 ( 1) 0.050 +- 0.000

6 D 1.00000 0.00000 0.00000 ( 1) 0.050 +- 0.000

7 Q 1.00000 0.00000 0.00000 ( 1) 0.050 +- 0.000

8 G 1.00000 0.00000 0.00000 ( 1) 0.050 +- 0.000

9 T 1.00000 0.00000 0.00000 ( 1) 0.050 +- 0.001

10 T 0.70559 0.23882 0.05560 ( 1) 0.358 +- 0.488

11 S 0.69079 0.24287 0.06634 ( 1) 0.377 +- 0.503

12 T 0.99998 0.00001 0.00000 ( 1) 0.050 +- 0.004

13 R 0.99981 0.00016 0.00003 ( 1) 0.050 +- 0.014

14 V 0.46747 0.41581 0.11672 ( 1) 0.615 +- 0.552

15 I 0.99996 0.00004 0.00001 ( 1) 0.050 +- 0.007

16 L 0.99883 0.00101 0.00016 ( 1) 0.051 +- 0.035

17 F 0.99985 0.00012 0.00003 ( 1) 0.050 +- 0.013

18 D 1.00000 0.00000 0.00000 ( 1) 0.050 +- 0.001

19 E 0.99869 0.00113 0.00018 ( 1) 0.051 +- 0.037

20 K 0.16089 0.65150 0.18761 ( 2) 0.942 +- 0.438

21 C 0.16203 0.69960 0.13837 ( 2) 0.916 +- 0.418

22 Q 0.99092 0.00820 0.00088 ( 1) 0.059 +- 0.096

23 S 0.00096 0.66158 0.33746 ( 2) 1.171 +- 0.260

24 I 0.95768 0.03274 0.00958 ( 1) 0.095 +- 0.222

25 H 0.94778 0.04595 0.00627 ( 1) 0.103 +- 0.228

26 T 0.36936 0.47220 0.15843 ( 2) 0.729 +- 0.549

27 E 0.98651 0.01142 0.00207 ( 1) 0.064 +- 0.121

28 Q 0.97452 0.02242 0.00306 ( 1) 0.076 +- 0.161

29 E 0.27883 0.53143 0.18974 ( 2) 0.831 +- 0.522

30 E 0.82158 0.15520 0.02322 ( 1) 0.231 +- 0.396

31 F 1.00000 0.00000 0.00000 ( 1) 0.050 +- 0.000

32 D 0.75684 0.20119 0.04197 ( 1) 0.302 +- 0.457

33 F 0.99767 0.00220 0.00014 ( 1) 0.052 +- 0.048

34 P 1.00000 0.00000 0.00000 ( 1) 0.050 +- 0.001

35 H 0.95120 0.04129 0.00750 ( 1) 0.100 +- 0.225

36 P 0.99961 0.00035 0.00005 ( 1) 0.050 +- 0.020

37 G 1.00000 0.00000 0.00000 ( 1) 0.050 +- 0.001

38 W 1.00000 0.00000 0.00000 ( 1) 0.050 +- 0.000

39 V 0.99989 0.00011 0.00000 ( 1) 0.050 +- 0.010

40 E 1.00000 0.00000 0.00000 ( 1) 0.050 +- 0.000

41 Q 1.00000 0.00000 0.00000 ( 1) 0.050 +- 0.000

42 D 1.00000 0.00000 0.00000 ( 1) 0.050 +- 0.000

43 P 1.00000 0.00000 0.00000 ( 1) 0.050 +- 0.000

44 E 0.42423 0.45217 0.12360 ( 2) 0.659 +- 0.547

45 V 1.00000 0.00000 0.00000 ( 1) 0.050 +- 0.001

46 I 1.00000 0.00000 0.00000 ( 1) 0.050 +- 0.000

47 Y 0.99796 0.00189 0.00015 ( 1) 0.052 +- 0.045

48 T 0.01761 0.59677 0.38562 ( 2) 1.198 +- 0.457

49 S 0.99733 0.00246 0.00021 ( 1) 0.053 +- 0.052

50 V 0.99474 0.00466 0.00059 ( 1) 0.055 +- 0.074

51 V 1.00000 0.00000 0.00000 ( 1) 0.050 +- 0.000

52 N 0.07684 0.71093 0.21223 ( 2) 1.034 +- 0.353

53 L 0.99996 0.00004 0.00000 ( 1) 0.050 +- 0.006

54 M 1.00000 0.00000 0.00000 ( 1) 0.050 +- 0.000

55 K 0.99999 0.00001 0.00000 ( 1) 0.050 +- 0.003

56 K 0.99999 0.00001 0.00000 ( 1) 0.050 +- 0.003

57 C 0.99847 0.00135 0.00018 ( 1) 0.052 +- 0.040

58 L 1.00000 0.00000 0.00000 ( 1) 0.050 +- 0.001

59 V 0.99999 0.00001 0.00000 ( 1) 0.050 +- 0.003

60 N 0.99858 0.00130 0.00012 ( 1) 0.051 +- 0.038

61 T 1.00000 0.00000 0.00000 ( 1) 0.050 +- 0.001

62 G 0.98921 0.00930 0.00148 ( 1) 0.061 +- 0.107

63 I 0.01226 0.67630 0.31143 ( 2) 1.148 +- 0.287

64 N 0.00067 0.57432 0.42501 ( 2) 1.225 +- 0.352

65 K 0.99989 0.00010 0.00001 ( 1) 0.050 +- 0.011

66 D 0.11199 0.64004 0.24797 ( 2) 1.019 +- 0.409

67 I 1.00000 0.00000 0.00000 ( 1) 0.050 +- 0.002

68 A 0.99510 0.00451 0.00039 ( 1) 0.055 +- 0.070

69 A 0.99999 0.00001 0.00000 ( 1) 0.050 +- 0.003

70 I 0.99981 0.00016 0.00002 ( 1) 0.050 +- 0.014

71 G 1.00000 0.00000 0.00000 ( 1) 0.050 +- 0.000

72 I 1.00000 0.00000 0.00000 ( 1) 0.050 +- 0.000

73 T 1.00000 0.00000 0.00000 ( 1) 0.050 +- 0.000

74 N 1.00000 0.00000 0.00000 ( 1) 0.050 +- 0.000

75 Q 1.00000 0.00000 0.00000 ( 1) 0.050 +- 0.000

76 R 1.00000 0.00000 0.00000 ( 1) 0.050 +- 0.000

77 E 1.00000 0.00000 0.00000 ( 1) 0.050 +- 0.000

78 T 1.00000 0.00000 0.00000 ( 1) 0.050 +- 0.000

79 T 0.99592 0.00369 0.00039 ( 1) 0.054 +- 0.064

80 V 1.00000 0.00000 0.00000 ( 1) 0.050 +- 0.000

81 M 0.99999 0.00001 0.00000 ( 1) 0.050 +- 0.003

82 W 1.00000 0.00000 0.00000 ( 1) 0.050 +- 0.000

83 D 1.00000 0.00000 0.00000 ( 1) 0.050 +- 0.000

84 K 1.00000 0.00000 0.00000 ( 1) 0.050 +- 0.002

85 R 0.00002 0.60781 0.39217 ( 2) 1.205 +- 0.316

86 T 1.00000 0.00000 0.00000 ( 1) 0.050 +- 0.000

87 G 1.00000 0.00000 0.00000 ( 1) 0.050 +- 0.001

88 K 1.00000 0.00000 0.00000 ( 1) 0.050 +- 0.000

89 P 1.00000 0.00000 0.00000 ( 1) 0.050 +- 0.000

90 I 1.00000 0.00000 0.00000 ( 1) 0.050 +- 0.000

91 Y 1.00000 0.00000 0.00000 ( 1) 0.050 +- 0.001

92 N 1.00000 0.00000 0.00000 ( 1) 0.050 +- 0.000

93 A 1.00000 0.00000 0.00000 ( 1) 0.050 +- 0.000

94 I 1.00000 0.00000 0.00000 ( 1) 0.050 +- 0.002

95 V 1.00000 0.00000 0.00000 ( 1) 0.050 +- 0.000

96 W 1.00000 0.00000 0.00000 ( 1) 0.050 +- 0.000

97 Q 0.99240 0.00686 0.00074 ( 1) 0.058 +- 0.088

98 S 1.00000 0.00000 0.00000 ( 1) 0.050 +- 0.000

99 K 0.99659 0.00309 0.00032 ( 1) 0.053 +- 0.059

100 Q 1.00000 0.00000 0.00000 ( 1) 0.050 +- 0.000

101 S 0.99994 0.00005 0.00000 ( 1) 0.050 +- 0.008

102 G 0.99992 0.00007 0.00001 ( 1) 0.050 +- 0.009

103 N 0.87919 0.09289 0.02792 ( 1) 0.180 +- 0.372

104 E 0.74778 0.21781 0.03441 ( 1) 0.307 +- 0.451

105 T 0.99836 0.00150 0.00014 ( 1) 0.052 +- 0.041

106 S 0.99752 0.00222 0.00026 ( 1) 0.052 +- 0.051

107 Y 0.89806 0.08514 0.01680 ( 1) 0.155 +- 0.319

108 L 0.99999 0.00001 0.00000 ( 1) 0.050 +- 0.002

109 M 0.99488 0.00478 0.00034 ( 1) 0.055 +- 0.071

110 E 0.99274 0.00624 0.00101 ( 1) 0.057 +- 0.088

111 K 0.99997 0.00003 0.00000 ( 1) 0.050 +- 0.006

112 I 0.86069 0.12774 0.01158 ( 1) 0.188 +- 0.347

113 F 1.00000 0.00000 0.00000 ( 1) 0.050 +- 0.000

114 Q 0.99133 0.00815 0.00052 ( 1) 0.058 +- 0.092

115 S 0.99974 0.00024 0.00002 ( 1) 0.050 +- 0.016

116 K 1.00000 0.00000 0.00000 ( 1) 0.050 +- 0.001

117 T 1.00000 0.00000 0.00000 ( 1) 0.050 +- 0.000

118 G 1.00000 0.00000 0.00000 ( 1) 0.050 +- 0.000

119 L 1.00000 0.00000 0.00000 ( 1) 0.050 +- 0.001

120 V 0.99996 0.00004 0.00000 ( 1) 0.050 +- 0.006

121 L 1.00000 0.00000 0.00000 ( 1) 0.050 +- 0.001

122 N 1.00000 0.00000 0.00000 ( 1) 0.050 +- 0.001

123 P 0.99999 0.00001 0.00000 ( 1) 0.050 +- 0.004

124 Y 1.00000 0.00000 0.00000 ( 1) 0.050 +- 0.000

125 F 1.00000 0.00000 0.00000 ( 1) 0.050 +- 0.000

126 S 0.99998 0.00002 0.00000 ( 1) 0.050 +- 0.004

127 A 1.00000 0.00000 0.00000 ( 1) 0.050 +- 0.000

128 S 1.00000 0.00000 0.00000 ( 1) 0.050 +- 0.001

129 K 1.00000 0.00000 0.00000 ( 1) 0.050 +- 0.000

130 I 0.99993 0.00006 0.00001 ( 1) 0.050 +- 0.008

131 M 0.99980 0.00019 0.00001 ( 1) 0.050 +- 0.014

132 W 1.00000 0.00000 0.00000 ( 1) 0.050 +- 0.000

133 I 1.00000 0.00000 0.00000 ( 1) 0.050 +- 0.001

134 F 0.99998 0.00002 0.00000 ( 1) 0.050 +- 0.005

135 N 0.99964 0.00033 0.00003 ( 1) 0.050 +- 0.019

136 N 1.00000 0.00000 0.00000 ( 1) 0.050 +- 0.000

137 V 0.99999 0.00000 0.00000 ( 1) 0.050 +- 0.002

138 E 0.34718 0.45333 0.19949 ( 2) 0.771 +- 0.562

139 G 0.71412 0.23042 0.05546 ( 1) 0.349 +- 0.485

140 A 1.00000 0.00000 0.00000 ( 1) 0.050 +- 0.002

141 K 0.99973 0.00024 0.00003 ( 1) 0.050 +- 0.017

142 A 0.98318 0.01493 0.00189 ( 1) 0.067 +- 0.131

143 L 0.99990 0.00010 0.00001 ( 1) 0.050 +- 0.010

144 A 0.98430 0.01407 0.00163 ( 1) 0.066 +- 0.126

145 E 0.97045 0.02639 0.00316 ( 1) 0.080 +- 0.172

146 E 0.99561 0.00388 0.00051 ( 1) 0.054 +- 0.068

147 G 1.00000 0.00000 0.00000 ( 1) 0.050 +- 0.000

148 V 0.99729 0.00233 0.00038 ( 1) 0.053 +- 0.054

149 N 1.00000 0.00000 0.00000 ( 1) 0.050 +- 0.000

150 L 1.00000 0.00000 0.00000 ( 1) 0.050 +- 0.000

151 T 1.00000 0.00000 0.00000 ( 1) 0.050 +- 0.000

152 G 0.99942 0.00053 0.00005 ( 1) 0.051 +- 0.024

153 G 1.00000 0.00000 0.00000 ( 1) 0.050 +- 0.000

154 H 1.00000 0.00000 0.00000 ( 1) 0.050 +- 0.001

155 T 1.00000 0.00000 0.00000 ( 1) 0.050 +- 0.000

156 D 1.00000 0.00000 0.00000 ( 1) 0.050 +- 0.000

157 I 1.00000 0.00000 0.00000 ( 1) 0.050 +- 0.001

158 S 1.00000 0.00000 0.00000 ( 1) 0.050 +- 0.002

159 N 1.00000 0.00000 0.00000 ( 1) 0.050 +- 0.000

160 A 1.00000 0.00000 0.00000 ( 1) 0.050 +- 0.000

161 A 0.99998 0.00001 0.00000 ( 1) 0.050 +- 0.004

162 R 1.00000 0.00000 0.00000 ( 1) 0.050 +- 0.000

163 T 1.00000 0.00000 0.00000 ( 1) 0.050 +- 0.000

164 L 1.00000 0.00000 0.00000 ( 1) 0.050 +- 0.002

165 L 1.00000 0.00000 0.00000 ( 1) 0.050 +- 0.000

166 F 1.00000 0.00000 0.00000 ( 1) 0.050 +- 0.000

167 N 1.00000 0.00000 0.00000 ( 1) 0.050 +- 0.001

168 I 1.00000 0.00000 0.00000 ( 1) 0.050 +- 0.001

169 Y 0.76366 0.16899 0.06735 ( 1) 0.311 +- 0.498

170 E 1.00000 0.00000 0.00000 ( 1) 0.050 +- 0.001

171 K 0.99669 0.00314 0.00017 ( 1) 0.053 +- 0.056

172 K 0.89953 0.08527 0.01519 ( 1) 0.153 +- 0.314

173 W 1.00000 0.00000 0.00000 ( 1) 0.050 +- 0.000

174 D 0.99975 0.00023 0.00002 ( 1) 0.050 +- 0.016

175 D 0.04721 0.70075 0.25204 ( 2) 1.082 +- 0.319

176 E 0.99999 0.00001 0.00000 ( 1) 0.050 +- 0.003

177 L 1.00000 0.00000 0.00000 ( 1) 0.050 +- 0.001

178 L 0.99990 0.00010 0.00000 ( 1) 0.050 +- 0.010

179 A 0.99999 0.00001 0.00000 ( 1) 0.050 +- 0.004

180 K 0.99995 0.00005 0.00000 ( 1) 0.050 +- 0.007

181 T 1.00000 0.00000 0.00000 ( 1) 0.050 +- 0.000

182 N 0.41641 0.49612 0.08748 ( 2) 0.648 +- 0.524

183 I 1.00000 0.00000 0.00000 ( 1) 0.050 +- 0.001

184 P 0.99999 0.00001 0.00000 ( 1) 0.050 +- 0.004

185 K 0.99992 0.00008 0.00000 ( 1) 0.050 +- 0.009

186 S 0.70044 0.24763 0.05192 ( 1) 0.362 +- 0.497

187 I 0.87885 0.09736 0.02379 ( 1) 0.177 +- 0.350

188 L 1.00000 0.00000 0.00000 ( 1) 0.050 +- 0.000

189 P 1.00000 0.00000 0.00000 ( 1) 0.050 +- 0.000

190 I 1.00000 0.00000 0.00000 ( 1) 0.050 +- 0.001

191 V 1.00000 0.00000 0.00000 ( 1) 0.050 +- 0.001

192 K 1.00000 0.00000 0.00000 ( 1) 0.050 +- 0.000

193 Q 0.82195 0.15712 0.02094 ( 1) 0.230 +- 0.392

194 S 1.00000 0.00000 0.00000 ( 1) 0.050 +- 0.002

195 S 0.99997 0.00003 0.00000 ( 1) 0.050 +- 0.005

196 D 0.91491 0.07529 0.00979 ( 1) 0.136 +- 0.285

197 D 0.99997 0.00003 0.00000 ( 1) 0.050 +- 0.006

198 F 1.00000 0.00000 0.00000 ( 1) 0.050 +- 0.000

199 G 1.00000 0.00000 0.00000 ( 1) 0.050 +- 0.000

200 I 0.01055 0.60970 0.37975 ( 2) 1.190 +- 0.345

201 V 1.00000 0.00000 0.00000 ( 1) 0.050 +- 0.001

202 S 0.79042 0.17404 0.03554 ( 1) 0.267 +- 0.432

203 T 0.96449 0.03059 0.00492 ( 1) 0.086 +- 0.192

204 I 0.99998 0.00002 0.00000 ( 1) 0.050 +- 0.004

205 Q 0.43878 0.44449 0.11674 ( 2) 0.642 +- 0.547

206 E 0.99976 0.00022 0.00002 ( 1) 0.050 +- 0.016

207 F 0.99999 0.00001 0.00000 ( 1) 0.050 +- 0.002

208 H 0.99999 0.00001 0.00000 ( 1) 0.050 +- 0.002

209 I 1.00000 0.00000 0.00000 ( 1) 0.050 +- 0.001

210 T 0.99934 0.00060 0.00005 ( 1) 0.051 +- 0.026

211 G 1.00000 0.00000 0.00000 ( 1) 0.050 +- 0.000

212 V 0.99288 0.00670 0.00042 ( 1) 0.057 +- 0.083

213 A 0.99999 0.00001 0.00000 ( 1) 0.050 +- 0.003

214 G 1.00000 0.00000 0.00000 ( 1) 0.050 +- 0.000

215 D 1.00000 0.00000 0.00000 ( 1) 0.050 +- 0.000

216 Q 1.00000 0.00000 0.00000 ( 1) 0.050 +- 0.000

217 Q 1.00000 0.00000 0.00000 ( 1) 0.050 +- 0.000

218 A 0.99969 0.00030 0.00001 ( 1) 0.050 +- 0.017

219 S 0.99999 0.00001 0.00000 ( 1) 0.050 +- 0.004

220 L 0.99979 0.00019 0.00001 ( 1) 0.050 +- 0.014

221 F 1.00000 0.00000 0.00000 ( 1) 0.050 +- 0.001

222 G 1.00000 0.00000 0.00000 ( 1) 0.050 +- 0.000

223 H 1.00000 0.00000 0.00000 ( 1) 0.050 +- 0.000

224 G 0.99995 0.00005 0.00000 ( 1) 0.050 +- 0.007

225 S 0.99967 0.00030 0.00003 ( 1) 0.050 +- 0.018

226 P 0.99904 0.00090 0.00006 ( 1) 0.051 +- 0.031

227 I 1.00000 0.00000 0.00000 ( 1) 0.050 +- 0.002

228 G 1.00000 0.00000 0.00000 ( 1) 0.050 +- 0.001

229 G 0.99997 0.00003 0.00000 ( 1) 0.050 +- 0.006

230 C 0.99997 0.00003 0.00000 ( 1) 0.050 +- 0.005

231 K 1.00000 0.00000 0.00000 ( 1) 0.050 +- 0.000

232 S 0.99999 0.00001 0.00000 ( 1) 0.050 +- 0.003

233 T 1.00000 0.00000 0.00000 ( 1) 0.050 +- 0.000

234 Y 1.00000 0.00000 0.00000 ( 1) 0.050 +- 0.000

235 G 1.00000 0.00000 0.00000 ( 1) 0.050 +- 0.000

236 T 1.00000 0.00000 0.00000 ( 1) 0.050 +- 0.000

237 G 1.00000 0.00000 0.00000 ( 1) 0.050 +- 0.000

238 C 0.99985 0.00014 0.00001 ( 1) 0.050 +- 0.012

239 F 1.00000 0.00000 0.00000 ( 1) 0.050 +- 0.000

240 V 0.99995 0.00005 0.00001 ( 1) 0.050 +- 0.007

241 V 1.00000 0.00000 0.00000 ( 1) 0.050 +- 0.001

242 K 0.99997 0.00002 0.00000 ( 1) 0.050 +- 0.005

243 N 1.00000 0.00000 0.00000 ( 1) 0.050 +- 0.000

244 I 0.99999 0.00001 0.00000 ( 1) 0.050 +- 0.002

245 G 1.00000 0.00000 0.00000 ( 1) 0.050 +- 0.000

246 D 0.01697 0.79893 0.18410 ( 2) 1.076 +- 0.237

247 T 1.00000 0.00000 0.00000 ( 1) 0.050 +- 0.001

248 I 0.82366 0.14739 0.02896 ( 1) 0.232 +- 0.402

249 K 0.99069 0.00843 0.00088 ( 1) 0.059 +- 0.097

250 E 0.99928 0.00069 0.00003 ( 1) 0.051 +- 0.026

251 I 1.00000 0.00000 0.00000 ( 1) 0.050 +- 0.001

252 P 0.99759 0.00212 0.00029 ( 1) 0.052 +- 0.050

253 K 0.99993 0.00007 0.00000 ( 1) 0.050 +- 0.008

254 G 1.00000 0.00000 0.00000 ( 1) 0.050 +- 0.000

255 L 1.00000 0.00000 0.00000 ( 1) 0.050 +- 0.000

256 L 1.00000 0.00000 0.00000 ( 1) 0.050 +- 0.000

257 A 0.88754 0.10185 0.01061 ( 1) 0.162 +- 0.319

258 T 1.00000 0.00000 0.00000 ( 1) 0.050 +- 0.000

259 V 0.99595 0.00361 0.00044 ( 1) 0.054 +- 0.065

260 G 1.00000 0.00000 0.00000 ( 1) 0.050 +- 0.001

261 W 1.00000 0.00000 0.00000 ( 1) 0.050 +- 0.001

262 E 1.00000 0.00000 0.00000 ( 1) 0.050 +- 0.000

263 I 0.99993 0.00007 0.00000 ( 1) 0.050 +- 0.008

264 N 0.99999 0.00001 0.00000 ( 1) 0.050 +- 0.003

265 G 0.99993 0.00007 0.00001 ( 1) 0.050 +- 0.008

266 K 0.99975 0.00023 0.00002 ( 1) 0.050 +- 0.016

267 I 0.83069 0.14727 0.02204 ( 1) 0.222 +- 0.387

268 T 0.84301 0.14023 0.01676 ( 1) 0.208 +- 0.370

269 Y 1.00000 0.00000 0.00000 ( 1) 0.050 +- 0.000

270 A 1.00000 0.00000 0.00000 ( 1) 0.050 +- 0.000

271 L 1.00000 0.00000 0.00000 ( 1) 0.050 +- 0.001

272 E 1.00000 0.00000 0.00000 ( 1) 0.050 +- 0.000

273 G 1.00000 0.00000 0.00000 ( 1) 0.050 +- 0.000

274 T 0.99980 0.00018 0.00002 ( 1) 0.050 +- 0.014

275 V 1.00000 0.00000 0.00000 ( 1) 0.050 +- 0.001

276 M 1.00000 0.00000 0.00000 ( 1) 0.050 +- 0.001

277 T 0.99993 0.00007 0.00001 ( 1) 0.050 +- 0.009

278 A 1.00000 0.00000 0.00000 ( 1) 0.050 +- 0.000

279 G 1.00000 0.00000 0.00000 ( 1) 0.050 +- 0.000

280 A 1.00000 0.00000 0.00000 ( 1) 0.050 +- 0.002

281 A 0.99998 0.00002 0.00000 ( 1) 0.050 +- 0.004

282 L 1.00000 0.00000 0.00000 ( 1) 0.050 +- 0.002

283 K 0.99958 0.00039 0.00003 ( 1) 0.050 +- 0.021

284 W 1.00000 0.00000 0.00000 ( 1) 0.050 +- 0.000

285 I 1.00000 0.00000 0.00000 ( 1) 0.050 +- 0.001

286 R 0.50149 0.35234 0.14617 ( 1) 0.597 +- 0.574

287 D 0.99997 0.00003 0.00000 ( 1) 0.050 +- 0.006

288 I 0.99668 0.00285 0.00047 ( 1) 0.053 +- 0.060

289 G 0.99957 0.00039 0.00004 ( 1) 0.050 +- 0.021

290 I 0.99999 0.00001 0.00000 ( 1) 0.050 +- 0.003

291 L 0.99998 0.00001 0.00000 ( 1) 0.050 +- 0.004

292 K 0.00226 0.74721 0.25052 ( 2) 1.124 +- 0.225

293 D 0.99999 0.00001 0.00000 ( 1) 0.050 +- 0.003

294 Y 0.91353 0.07769 0.00878 ( 1) 0.137 +- 0.285

295 N 0.84981 0.12436 0.02583 ( 1) 0.206 +- 0.378

296 E 1.00000 0.00000 0.00000 ( 1) 0.050 +- 0.000

297 I 0.88949 0.09672 0.01379 ( 1) 0.162 +- 0.323

298 S 0.98443 0.01432 0.00125 ( 1) 0.065 +- 0.124

299 K 0.61552 0.32502 0.05946 ( 1) 0.445 +- 0.513

300 I 1.00000 0.00000 0.00000 ( 1) 0.050 +- 0.001

301 V 1.00000 0.00000 0.00000 ( 1) 0.050 +- 0.001

302 T 0.99990 0.00010 0.00001 ( 1) 0.050 +- 0.010

303 S 0.04218 0.71609 0.24173 ( 2) 1.083 +- 0.315

304 K 0.99790 0.00189 0.00021 ( 1) 0.052 +- 0.046

305 N 1.00000 0.00000 0.00000 ( 1) 0.050 +- 0.001

306 G 0.07197 0.74737 0.18066 ( 2) 1.022 +- 0.332

307 G 1.00000 0.00000 0.00000 ( 1) 0.050 +- 0.001

308 V 1.00000 0.00000 0.00000 ( 1) 0.050 +- 0.001

309 Y 0.99997 0.00003 0.00000 ( 1) 0.050 +- 0.006

310 F 1.00000 0.00000 0.00000 ( 1) 0.050 +- 0.000

311 V 1.00000 0.00000 0.00000 ( 1) 0.050 +- 0.000

312 P 1.00000 0.00000 0.00000 ( 1) 0.050 +- 0.000

313 A 1.00000 0.00000 0.00000 ( 1) 0.050 +- 0.000

314 F 1.00000 0.00000 0.00000 ( 1) 0.050 +- 0.000

315 Q 0.95925 0.03720 0.00355 ( 1) 0.091 +- 0.199

316 G 1.00000 0.00000 0.00000 ( 1) 0.050 +- 0.000

317 L 1.00000 0.00000 0.00000 ( 1) 0.050 +- 0.000

318 G 0.99936 0.00059 0.00005 ( 1) 0.051 +- 0.025

319 T 1.00000 0.00000 0.00000 ( 1) 0.050 +- 0.002

320 P 1.00000 0.00000 0.00000 ( 1) 0.050 +- 0.000

321 Y 0.99989 0.00011 0.00001 ( 1) 0.050 +- 0.010

322 W 1.00000 0.00000 0.00000 ( 1) 0.050 +- 0.000

323 D 0.99996 0.00004 0.00000 ( 1) 0.050 +- 0.006

324 D 0.99993 0.00006 0.00000 ( 1) 0.050 +- 0.008

325 D 1.00000 0.00000 0.00000 ( 1) 0.050 +- 0.002

326 V 1.00000 0.00000 0.00000 ( 1) 0.050 +- 0.000

327 R 1.00000 0.00000 0.00000 ( 1) 0.050 +- 0.000

328 G 1.00000 0.00000 0.00000 ( 1) 0.050 +- 0.000

329 I 0.99829 0.00159 0.00013 ( 1) 0.052 +- 0.041

330 I 1.00000 0.00000 0.00000 ( 1) 0.050 +- 0.000

331 V 0.88026 0.10189 0.01785 ( 1) 0.173 +- 0.339

332 G 1.00000 0.00000 0.00000 ( 1) 0.050 +- 0.000

333 L 1.00000 0.00000 0.00000 ( 1) 0.050 +- 0.000

334 T 1.00000 0.00000 0.00000 ( 1) 0.050 +- 0.002

335 S 0.98471 0.01439 0.00090 ( 1) 0.065 +- 0.121

336 G 0.99989 0.00010 0.00001 ( 1) 0.050 +- 0.010

337 T 1.00000 0.00000 0.00000 ( 1) 0.050 +- 0.000

338 G 0.61572 0.29797 0.08631 ( 1) 0.459 +- 0.534

339 K 0.99903 0.00088 0.00009 ( 1) 0.051 +- 0.031

340 G 0.03171 0.71990 0.24839 ( 2) 1.095 +- 0.294

341 E 1.00000 0.00000 0.00000 ( 1) 0.050 +- 0.000

342 L 1.00000 0.00000 0.00000 ( 1) 0.050 +- 0.001

343 V 1.00000 0.00000 0.00000 ( 1) 0.050 +- 0.000

1. **Lambda light chain antibody variable region: mlc file**

CODONML (in paml version 4, June 2007) seq.nuc Model: One dN/dS ratio

Codon frequencies: F3x4

Site-class models: PositiveSelection

ns = 9 ls = 80

TREE # 1: (1, ((2, (((4, 6), 7), 8)), 9), (3, 5)); MP score: 161

lnL(ntime: 15 np: 20): -1133.338944 +0.000000

10..1 10..11 11..12 12..2 12..13 13..14 14..15 15..4 15..6 14..7 13..8 11..9 10..16 16..3 16..5

0.095373 0.000000 0.000000 0.499281 0.000000 0.065914 0.249295 0.781961 0.760671 0.211007 0.066703 0.000004 1.151299 0.000004 0.223316 1.624926 0.608880 0.341507 0.041623 4.641113

Note: Branch length is defined as number of nucleotide substitutions per codon (not per neucleotide site).

tree length = 4.10483

(1: 0.095373, ((2: 0.499281, (((4: 0.781961, 6: 0.760671): 0.249295, 7: 0.211007): 0.065914, 8: 0.066703): 0.000000): 0.000000, 9: 0.000004): 0.000000, (3: 0.000004, 5: 0.223316): 1.151299);

(1: 0.095373, ((2: 0.499281, (((6: 0.781961, 8: 0.760671): 0.249295, 9: 0.211007): 0.065914, 3: 0.066703): 0.000000): 0.000000, 5: 0.000004): 0.000000, (4: 0.000004, 7: 0.223316): 1.151299);

Detailed output identifying parameters

kappa (ts/tv) = 1.62493

dN/dS for site classes (K=3)

p: 0.60888 0.34151 0.04961

w: 0.04162 1.00000 4.64111

dN & dS for each branch

branch t N S dN/dS dN dS N*dN S*dS

10..1 0.095 181.3 58.7 0.5971 0.0273 0.0457 4.9 2.7

10..11 0.000 181.3 58.7 0.5971 0.0000 0.0000 0.0 0.0

11..12 0.000 181.3 58.7 0.5971 0.0000 0.0000 0.0 0.0

12..2 0.499 181.3 58.7 0.5971 0.1428 0.2392 25.9 14.0

12..13 0.000 181.3 58.7 0.5971 0.0000 0.0000 0.0 0.0

13..14 0.066 181.3 58.7 0.5971 0.0189 0.0316 3.4 1.9

14..15 0.249 181.3 58.7 0.5971 0.0713 0.1194 12.9 7.0

15..4 0.782 181.3 58.7 0.5971 0.2237 0.3747 40.6 22.0

15..6 0.761 181.3 58.7 0.5971 0.2176 0.3645 39.5 21.4

14..7 0.211 181.3 58.7 0.5971 0.0604 0.1011 10.9 5.9

13..8 0.067 181.3 58.7 0.5971 0.0191 0.0320 3.5 1.9

11..9 0.000 181.3 58.7 0.5971 0.0000 0.0000 0.0 0.0

10..16 1.151 181.3 58.7 0.5971 0.3294 0.5516 59.7 32.4

16..3 0.000 181.3 58.7 0.5971 0.0000 0.0000 0.0 0.0

16..5 0.223 181.3 58.7 0.5971 0.0639 0.1070 11.6 6.3

Bayes Empirical Bayes (BEB) analysis (Yang, Wong & Nielsen 2005. Mol. Biol. Evol. 22:1107-1118)

Positively selected sites (*: P>95%; **: P>99%)

Pr(w>1) post mean +- SE for w

74 Y 0.859 5.090 +- 2.652

75 G 0.973* 5.768 +- 2.390

77 S 0.867 5.352 +- 2.728

1. **Lambda light chain antibody rst file**

lnL = -1133.338944

Bayes Empirical Bayes (BEB) probabilities for 3 classes (class)& postmean_w

1 used as reference

1 R 0.72360 0.27559 0.00082 ( 1) 0.314 +- 0.427

2 A 0.28706 0.71060 0.00234 ( 2) 0.730 +- 0.434

3 T 0.99548 0.00452 0.00001 ( 1) 0.055 +- 0.064

4 L 0.19448 0.80131 0.00422 ( 2) 0.820 +- 0.388

5 S 0.00759 0.97661 0.01580 ( 2) 1.020 +- 0.277

6 C 0.99612 0.00387 0.00000 ( 1) 0.054 +- 0.059

7 R 0.68383 0.31439 0.00178 ( 1) 0.353 +- 0.450

8 A 0.88386 0.11592 0.00022 ( 1) 0.161 +- 0.305

9 S 0.99505 0.00495 0.00001 ( 1) 0.055 +- 0.067

10 Q 0.97259 0.02737 0.00005 ( 1) 0.076 +- 0.155

11 S 0.31026 0.68724 0.00250 ( 2) 0.708 +- 0.445

12 V 0.00767 0.98246 0.00987 ( 2) 1.006 +- 0.180

13 S 0.13462 0.85840 0.00698 ( 2) 0.883 +- 0.364

14 S 0.02686 0.96443 0.00871 ( 2) 0.988 +- 0.234

15 Y 0.84748 0.15224 0.00028 ( 1) 0.196 +- 0.342

16 L 0.91884 0.08083 0.00032 ( 1) 0.128 +- 0.261

17 A 0.99440 0.00560 0.00001 ( 1) 0.056 +- 0.071

18 W 0.98102 0.01895 0.00003 ( 1) 0.068 +- 0.130

19 Y 0.99122 0.00877 0.00001 ( 1) 0.059 +- 0.089

20 Q 0.97181 0.02814 0.00005 ( 1) 0.077 +- 0.158

21 Q 0.96503 0.03489 0.00008 ( 1) 0.084 +- 0.175

22 K 0.74499 0.25438 0.00063 ( 1) 0.293 +- 0.415

23 P 0.96167 0.03824 0.00009 ( 1) 0.087 +- 0.183

24 G 0.98673 0.01325 0.00002 ( 1) 0.063 +- 0.109

25 Q 0.00439 0.97375 0.02186 ( 2) 1.036 +- 0.323

26 A 0.77572 0.22364 0.00064 ( 1) 0.264 +- 0.399

27 P 0.98848 0.01150 0.00001 ( 1) 0.061 +- 0.102

28 R 0.13303 0.86200 0.00497 ( 2) 0.879 +- 0.338

29 L 0.98883 0.01115 0.00001 ( 1) 0.061 +- 0.100

30 L 0.98271 0.01727 0.00002 ( 1) 0.067 +- 0.124

31 I 0.99629 0.00371 0.00000 ( 1) 0.054 +- 0.058

32 Y 0.72567 0.27358 0.00075 ( 1) 0.312 +- 0.426

33 G 0.00001 0.80495 0.19504 ( 2) 1.638 +- 1.493

34 A 0.94174 0.05807 0.00019 ( 1) 0.106 +- 0.224

35 S 0.99296 0.00704 0.00001 ( 1) 0.057 +- 0.080

36 T 0.00005 0.90450 0.09545 ( 2) 1.310 +- 1.118

37 R 0.00070 0.95085 0.04845 ( 2) 1.107 +- 0.546

38 A 0.00003 0.86652 0.13346 ( 2) 1.388 +- 1.138

39 T 0.00045 0.96392 0.03563 ( 2) 1.080 +- 0.497

40 G 0.75172 0.24746 0.00082 ( 1) 0.287 +- 0.414

41 I 0.01658 0.97693 0.00649 ( 2) 0.992 +- 0.170

42 P 0.94089 0.05891 0.00020 ( 1) 0.107 +- 0.226

43 D 0.00003 0.85930 0.14067 ( 2) 1.477 +- 1.373

44 R 0.98713 0.01285 0.00002 ( 1) 0.063 +- 0.107

45 F 0.99475 0.00525 0.00000 ( 1) 0.055 +- 0.069

46 S 0.99571 0.00429 0.00000 ( 1) 0.055 +- 0.063

47 G 0.99559 0.00440 0.00000 ( 1) 0.055 +- 0.063

48 S 0.99505 0.00495 0.00001 ( 1) 0.055 +- 0.067

49 G 0.96017 0.03973 0.00010 ( 1) 0.088 +- 0.187

50 S 0.98083 0.01914 0.00003 ( 1) 0.069 +- 0.131

51 G 0.96791 0.03203 0.00007 ( 1) 0.081 +- 0.168

52 T 0.94737 0.05246 0.00017 ( 1) 0.101 +- 0.214

53 D 0.01572 0.97795 0.00633 ( 2) 0.992 +- 0.158

54 F 0.99475 0.00525 0.00000 ( 1) 0.055 +- 0.069

55 I 0.77984 0.21948 0.00069 ( 1) 0.260 +- 0.397

56 L 0.99113 0.00886 0.00001 ( 1) 0.059 +- 0.089

57 T 0.99094 0.00905 0.00001 ( 1) 0.059 +- 0.090

58 I 0.99685 0.00315 0.00000 ( 1) 0.053 +- 0.054

59 S 0.99828 0.00171 0.00000 ( 1) 0.052 +- 0.040

60 R 0.02869 0.96202 0.00929 ( 2) 0.987 +- 0.245

61 L 0.92298 0.07672 0.00030 ( 1) 0.124 +- 0.255

62 E 0.00495 0.98281 0.01224 ( 2) 1.012 +- 0.194

63 P 0.09990 0.89358 0.00652 ( 2) 0.913 +- 0.313

64 E 0.97367 0.02629 0.00004 ( 1) 0.075 +- 0.152

65 D 0.99117 0.00882 0.00001 ( 1) 0.059 +- 0.089

66 F 0.07180 0.92081 0.00740 ( 2) 0.941 +- 0.280

67 A 0.94174 0.05807 0.00019 ( 1) 0.106 +- 0.224

68 V 0.00000 0.66233 0.33767 ( 2) 2.332 +- 2.213

69 Y 0.98173 0.01824 0.00003 ( 1) 0.068 +- 0.128

70 Y 0.99379 0.00620 0.00001 ( 1) 0.056 +- 0.075

71 C 0.98793 0.01205 0.00001 ( 1) 0.062 +- 0.104

72 Q 0.96503 0.03489 0.00008 ( 1) 0.084 +- 0.175

73 Q 0.96905 0.03089 0.00006 ( 1) 0.080 +- 0.165

74 Y 0.00000 0.14060 0.85940 ( 3) 5.090 +- 2.652

75 G 0.00000 0.02680 0.97320 ( 3) 5.768 +- 2.390

76 S 0.00195 0.96668 0.03137 ( 2) 1.089 +- 0.616

77 S 0.00000 0.13296 0.86704 ( 3) 5.352 +- 2.728

78 P 0.00870 0.97735 0.01395 ( 2) 1.020 +- 0.332

79 G 0.00016 0.81183 0.18800 ( 2) 1.815 +- 1.982

80 F 0.00039 0.96188 0.03772 ( 2) 1.094 +- 0.578
